# Transcriptome Analysis of the Inhibitory Effect of Astaxanthin on *Helicobacter pylori*-Induced Gastric Carcinoma Cell Motility

**DOI:** 10.3390/md18070365

**Published:** 2020-07-15

**Authors:** Suhn Hyung Kim, Hyeyoung Kim

**Affiliations:** Department of Food and Nutrition, Brain Korea 21 PLUS Project, College of Human Ecology, Yonsei University, Seoul 03722, Korea; cigdoli2@naver.com

**Keywords:** *Helicobacter pylori*, gastric carcinoma, astaxanthin, cell motility, cell migration

## Abstract

*Helicobacter pylori* (*H. pylori*) infection promotes the metastasis of gastric carcinoma cells by modulating signal transduction pathways that regulate cell proliferation, motility, and invasion. Astaxanthin (ASTX), a xanthophyll carotenoid, is known to inhibit cancer cell migration and invasion, however the mechanism of action of ASTX in *H. pylori*-infected gastric epithelial cells is not well understood. To gain insight into this process, we carried out a comparative RNA sequencing (RNA-Seq) analysis of human gastric cancer AGS (adenocarcinoma gastric) cells as a function of *H. pylori* infection and ASTX administration. The results were used to identify genes that are differently expressed in response to *H. pylori* and ASTX. Gene ontology (GO) analysis identified differentially expressed genes (DEGs) to be associated with cell cytoskeleton remodeling, motility, and/or migration. Among the 20 genes identified, those encoding c-MET, PI3KC2, PLCγ1, Cdc42, and ROCK1 were selected for verification by real-time PCR analysis. The verified genes were mapped, using signaling networks contained in the KEGG database, to create a signaling pathway through which ASTX might mitigate the effects of *H. pylori*-infection. We propose that *H. pylori*-induced upregulation of the upstream regulator c-MET, and hence, its downstream targets Cdc42 and ROCK1, is suppressed by ASTX. ASTX is also suggested to counteract *H. pylori*-induced activation of PI3K and PLCγ. In conclusion, ASTX can suppress *H. pylori*-induced gastric cancer progression by inhibiting cytoskeleton reorganization and reducing cell motility through downregulation of c-MET, EGFR, PI3KC2, PLCγ1, Cdc42, and ROCK1.

## 1. Introduction

Globally, gastric cancer is the fifth most common type of cancer and the third leading cause of death from cancer [1]. *Helicobacter pylori* infection is the major cause of gastric cancer, accounting for more than 60% of the cases [2]. Gastric cancer metastasizes at a high rate, localizing primarily in the liver, peritoneum, lung, and bone [3].

Metastatic cancer occurs as a result of the invasion of the surrounding stroma by primary tumor cells, followed by intravasation of the primary tumor into lymphatic or blood vessels, and finally, by extravasation and colonization of the tumor cells at a distant organ site [4]. Metastasis is initiated when cancer cells acquire specialized behaviors that allow them to breach the basement membrane of the extracellular matrix (ECM). Cancer cells undergo epithelia–mesenchymal transition (EMT), which enables them to lose intercellular junctions and to gain a migratory cell phenotype [5]. Modulation of cell motility is the determinant step in early local invasion. Cell motility requires continuous turnover of the cytoskeleton and modification of cell–cell and cell–substratum adhesions [6]. 

Cell locomotion involves three major processes. At the leading edge, the cell extends itself via reorganization of the actin cytoskeleton. At the trailing edge, the cell remodels its cytoskeleton to generate a contractile force. During this process, extracellular proteases are secreted to degrade the ECM, and to clear the path ahead [7]. A motile cell elongates itself at the leading edge through the polymerization of actin monomers into sheet-like structures known as lamellipodia. Protruding from the lamellipodial actin network are filopodia, which are spike-like structures formed from bundles of cross-linked actin microfilaments. Filopodia function in environmental sensing, cell migration, and cell–cell interaction. During the course of cell movement, the fliopodia located at the leading edge of the cell makes integrin to contact with the ECM while the cell breaks its surface adhesions at the rear, detaching its trailing edge from the substratum, and moving forward via an actomysin contractile force [8]. Increased filopodia formation and upregulation of filopodia proteins such as fascin and myosin-X promote cell migration, and are characteristic of invasive carcinoma cells [9]. 

Cell motility and morphology are regulated by small G proteins of the Rho family of the Ras-related GTPases. The Rho GTPases Rho, Rac, and cell division control protein 42 (Cdc42) coordinate the different steps of cell locomotion. Rho regulates formation of stress fibers and generation of the contractile force at the trailing edge of the moving cell. Rac modulates formation of membrane ruffles and lamellipodia, whereas Cdc42 is involved in formation of filopodia and focal adhesions at the leading edge of the cell [10]. Rho acts through its effector protein Rho-associated coiled-coil forming kinase (ROCK) to activate myosin light chain (MLC) for formation of stress fibers. Cdc42 and Rac regulate actin polymerization by activating Wiskott–Aldrich syndrome protein (WASP) and WASP-family verprolin-homologous protein (WAVE), respectively. WAVE and WASP activate the complex of actin-related proteins (ARP)2 and ARP3 to initiate the formation of new actin polymerization sites at lamellipodia. [11] 

The cell motility cascade mediated by Rho GTPases is activated by chemokines, cytokines, and growth factors. Chemokines such as chemokine C-X-C motif ligand 12 (CXCL12), and growth factors such as epidermal growth factor (EGF), hepatocyte growth factor (HGF), platelet-derived growth factor (PDGF), or fibroblast growth factor (FGF), bind cell surface receptors which activate signaling pathways that regulate cytoskeleton restructuring and cell migration [6,12]. Receptor tyrosine kinases that are activated upon binding their ligand growth factors, mediate the cell migration pathway. In particular, the EGR receptor (EGFR) and HGF receptor c-MET are highly expressed in many carcinomas [13,14]. 

In an early study, transcriptional array analysis of AGS cells infected with *H. pylori* indicated that genes associated the innate immune response (including the inflammatory response) and cell motility are the genes most impacted by the *H. pylori* infection [15]. *H. pylori* infection of the stomach lining promotes gastric carcinogenesis through increased inflammatory responses which lead to increased cell proliferation and migration [16,17,18]. *H. pylori* adhesion to gastric epithelial cells induces tyrosine kinase phosphorylation and results in cytoskeleton rearrangement [19]. *H. pylori*-induced upregulation of pro-inflammatory cytokines has been shown to promote cell motility and cell elongation [20]. In addition, *H. pylori* infection cause EMT by increasing soluble heparin-binding epidermal growth factor (HB-GF) shedding via upregulation of gastrin and matrix metalloprotease (MMP)-7 [21].

Virulent strains of *H. pylori* possess a cytotoxin-associated gene (cag) pathogenicity island (cagPAI) that encodes a type IV secretion system through which the virulence factor CagA is translocated into the host cell. Investigations have demonstrated that cagPAI is responsible for the upregulation of the proinflammatory cytokine interleukin-8 (IL-8), activation of EGFR and c-MET signaling, activation of the transcription factors activator protein-1 (AP-1) and nuclear factor kappa-light-chain-enhancer of activated B cells (NF-κB), activation of mitogen-activated protein (MAP) kinases and cellular stress response kinases, and activation of the small GTPases Rac1 and Cdc42 [22,23,24,25,26,27,28,29,30,31]. 

Astaxanthin (ASTX), a 3,30-dihydroxy-β, β-carotene-4,40-dione, is a xanthophyll carotenoid, produced primarily by marine microalgae/phytoplankton. ASTX gives lobster, salmon, and krill their red color [32]. ASTX’s multiple conjugated π-bonds give rise to ASTX’s powerful antioxidant properties [33], which underlie its cytoprotective effects [34,35]. Numerous studies have been carried out to identify and delineate the effects of ASTX on inflammation-driven diseases including cancer. Specifically, ASTX has been shown to inhibit the migration, invasion, and/or proliferation of cancerous gastric [36], liver [37], ovarian [38], breast [39], colon [40], and melanoma cells [41]. In addition, ASTX is known to target cancer-related signal transduction pathway proteins and their regulators, namely inflammatory cytokines, membrane receptors (e.g., peroxisome proliferator-activated receptor gamma (PPARγ), transcriptional regulators (e.g., NF-κB, signal transducer and activator of transcription 3 (STAT3), NF-2-related factor 2 (Nf2), and zinc finger E-box binding homeobox (ZEB)), and protein kinases (e.g., janus kinase (JAK), protein kinase B (PKB), phosphoinositide 3-kinase (PI3K) and the MAP kinases c-Jun *N*-terminal kinase (JNK), extracellular signal-related kinase (ERK), and p38) [35,36,37,38,39,40,41,42,43]. 

The present study was carried out to identify the cell signaling pathway(s) through which ASTX acts on gastric carcinoma AGS cells to mitigate *H. pylori*-induced activation of cytoskeleton remodeling, cell motility, and cell migration. RNA-sequencing (RNA-Seq) analysis was performed to determine which genes undergo altered expression in AGS cells in response to ASTX administration and *H-pylori*-infection. Gene ontology (GO) analysis identified differentially expressed genes (DEGs) to be associated with cell cytoskeleton remodeling, motility, and migration. Among these are DEGs encoding c-MET, PI3KC2β, PLCγ1, Cdc42, and ROCK1, which we confirmed by executing real-time PCR analysis. Herein, we propose a signaling-network through which ASTX might act to counter the effects of *H. pylori* infection, and thereby protect against *H. pylori*-induced gastric cancer metastasis.

## 2. Results

### 2.1. Gene Expression Profile of H. pylori-Infected and/or ASTX-Treated AGS Cells

**Figure 1:**
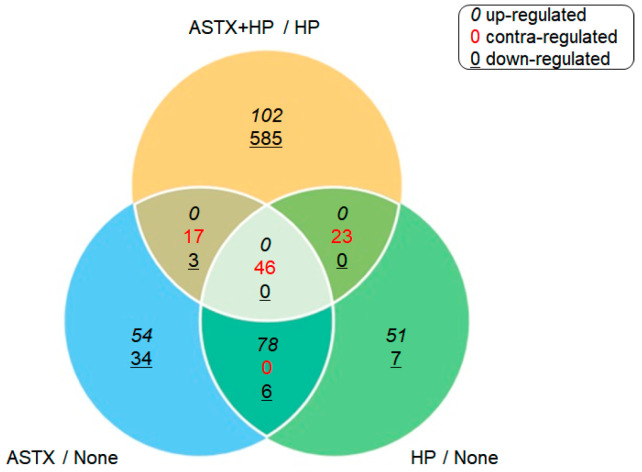
A comparison of the differentially expressed genes (DEG) expression pattern observed between pairs, and between paired-pairs, of the four experimental groups (None, *Helicobacter pylori*-infected AGS cells (HP), Astaxanthin (ASTX), and ASTX + HP). The numbers reported in black italic font correspond to upregulated DEGs, the numbers reported in black underlined font correspond to downregulated genes and the numbers reported in red regular font correspond to genes that are upregulated in one set of experimental groups and downregulated in the other.

Gene expression was compared between uninfected AGS cells (None) vs. *H. pylori*-infected AGS cells (HP), between HP and *H. pylori*-infected AGS cells treated with ASTX (ASTX + HP), and between None and uninfected AGS cells treated with ASTX (ASTX). RNA-Seq analysis identified 1006 DEGs among the four experimental systems. *H. pylori* infection upregulated 191 genes and downregulated 20 genes, whereas ASTX treatment upregulated 190 genes and downregulated 48 genes. ASTX treatment of the AGS cells prior to *H. pylori* infection resulted in upregulation of 112 genes, seven of which are repressed by *H. pylori* infection, and downregulation of 664 genes, 62 of which are upregulated in response to *H. pylori* infection. The DEGs observed for pairs of the four experimental groups are compared in the Venn diagram shown in Figure 1. 

### 2.2. GO Analysis of DEGs

**Figure 2:**
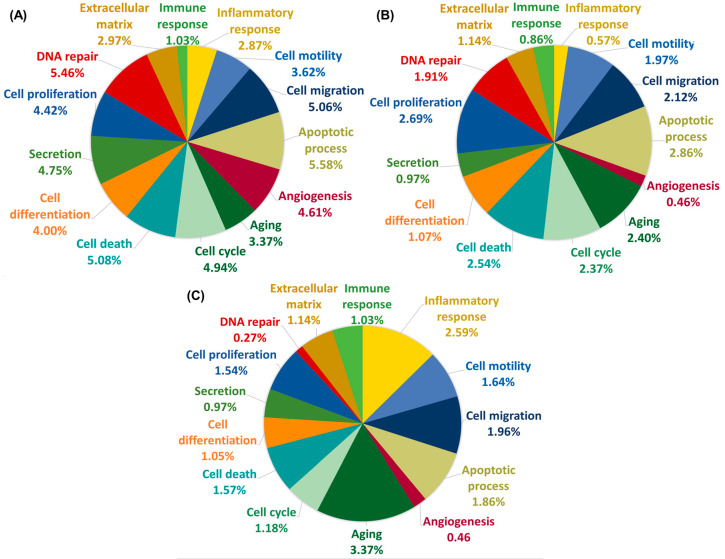
Pie chart representations of the findings from Gene Ontology (GO) biological process annotation analysis of DEGs associated with None vs. HP (**A**), or HP vs. ASTX + HP (**B**), or None vs. ASTX (**C**). The percent fraction of the DEGs annotated with a particular GO biological process is shown for each of the three pairs of experimental systems compared.

The DEGs identified above were further analyzed using GO annotation to identify and categorize each gene according to the respective biological process with which they are associated, namely aging, angiogenesis, apoptotic process, cell cycle, cell death, cell differentiation, cell migration, cell proliferation, DNA repair, extracellular matrix, immune response, inflammatory response, cell motility, and cell secretion. The GO categories shared between the DEGs observed for the experimental systems None and HP are inflammatory response, aging, cell migration, and cell motility (Figure 2A). The GO annotations of DEGs that are differentially regulated in the ASTX + HP vs. HP experimental systems include immune response, apoptotic process, cell migration, and DNA repair (Figure 2B). Genes differentially expressed by ASTX treatment in the absence of *H. pylori* infection are associated with cell proliferation, cell migration, the apoptotic process, and the cell cycle (Figure 2C). Among the DEGs that are strongly upregulated by ASTX treatment are genes that encode superoxide dismutase 1, superoxide dismutase 2, TNF receptor-associated factor 7, and serine/threonine kinase 17a (which regulates cell death and apoptosis), protein kinase N2, protein lipase C gamma (PLCγ), protein phosphatase 2, and ROCK1 (which regulates cell migration and cell death). 

**Figure 3:**
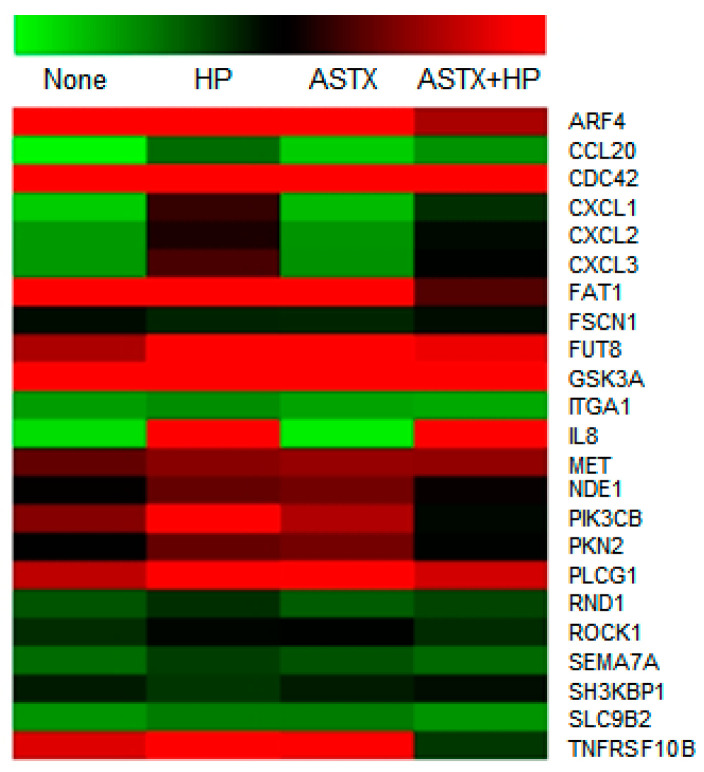
Heatmap representation of the expression levels of DEGs associated with cell migration/motility, which are upregulated in AGS cells in response to *H. pylori* infection but experience less, or no upregulation, in infected cells pretreated with ASTX. The normalized expression levels are reported in Table 1. The color gradient from green to red corresponds to increasing gene expression.

The overlap in the biological processes identified by the GO annotation analysis represented in Figure 2 centers on cell proliferation, apoptosis, and migration/motility. As the combined cell migration and cell motility categories make up the largest fraction of shared DEG annotations, we carried out further analysis of the DEGs that fall in these two categories. We were particularly interested in identifying the DEGs within this combined category that are upregulated in AGS cells in response to *H. pylori* infection but are not upregulated in AGS cells treated with ASTX prior to *H. pylori* infection. Accordingly, the normalized (log2) read counts determined for the DEGs of interest are reported in Table 1, and depicted in the heatmap shown in Figure 3. These results were used to construct a signal transduction pathway that governs cell migration/mobility and that is upregulated in AGS cells in response to *H. pylori* infection but not upregulated or upregulated to a smaller extent in cells treated with ASTX prior to infection. This pathway is presented and discussed in the following section. 

### 2.3. Functional Pathway Analysis of DEGs Altered by H. pylori Infection but Normalized by ASTX Pretreatment

**Figure 4:**
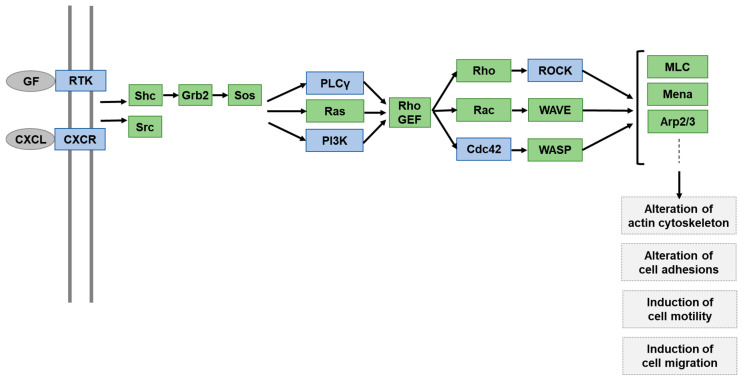
Depiction of a cancer cell motility and migration signaling network model that includes DEGs identified by RNA-seq analysis (Table 1) as displaying upregulated genes in *H. pylori*-infected in AGS cells. The DEG-encoded proteins are colored blue and the suggested pathway mediators are colored green.

Having identified the DEGs associated with cell motility and migration that are protected by ASTX from upregulation by *H. pylori* infection, our next step was to map them onto a signal transduction network, which we generated using the KEGG database and input from published studies of cell motility/migration signaling pathways. As illustrated in Figure 4, signaling is initiated by cytokine CXCL-mediated activation of the chemokine receptor CXCR, or by growth factor-mediated activation of a receptor tyrosine kinase. The cytokines CXCL1, CXCL2, and CXCL3 are among the DEGs through which ASTX mitigates *H. pylori*-induced upregulation (Table 1). In addition, the MET gene, which encodes the receptor tyrosine kinase c-MET (also known as HGF receptor), is also subject to upregulation by *H. pylori* and partially suppressed by AGS cell pretreatment with ASTX.

DEGs listed in Table 1 that impact downstream cell motility/migration signaling are PLCG1 (encodes phospholipase C gamma 1 (PLCγ1)), PIK3CB (encodes phosphatidylinositol-4,5-bisphosphate 3-kinase C2β (PI3KC2β)), CDC42 (encodes cell division control protein homolog 42 (Cdc42)), and ROCK1 (encodes Rho-associated coiled-coil containing protein kinase 1 (ROCK)). PI3KC2β and PLCγ1 have been implicated in the promotion of cytoskeleton rearrangement and cell motility [36]. Cdc42 and ROCK1 serve to transmit signals from PI3K and PLCγ to downstream partners. As is described under Section 2.4, c-MET, PI3KC2β, PLCγ1, Cdc42, and ROCK1 mRNA levels were determined to verify the RNA-seq results, and to compare the effects of *H. pylori* infection, and ASTX pretreatment, on the level of transcription. 

### 2.4. Determinations of c-MET, PI3KC2β, PLCγ1, Cdc42, and ROCK1 mRNA Levels in H. pylori-Infected AGS Cells, with and without ASTX Pretreatment 

**Figure 5:**
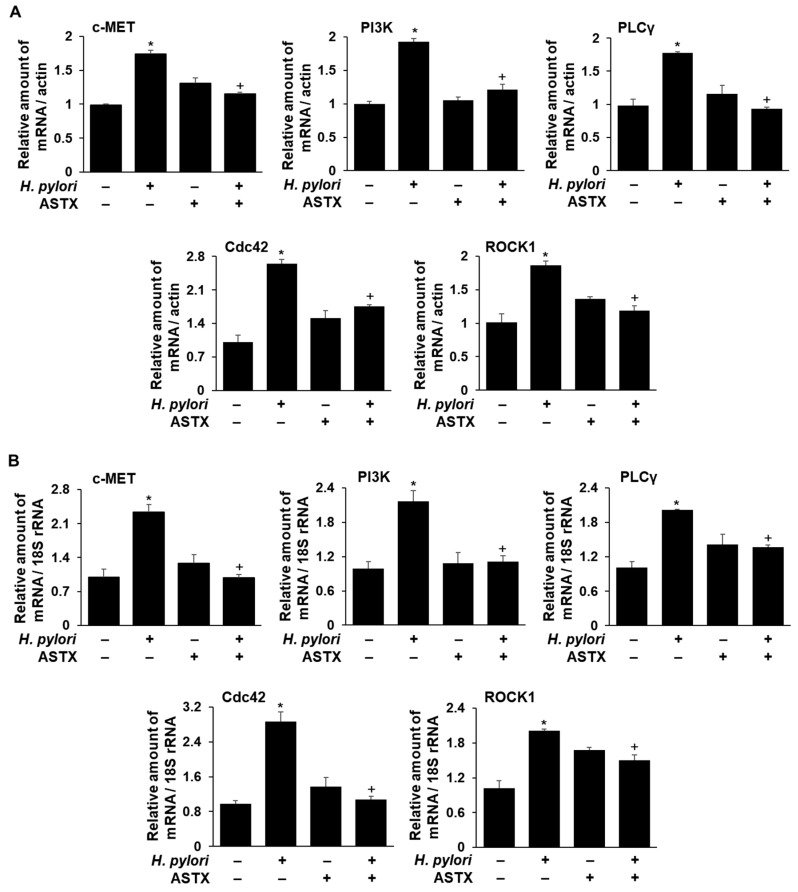
Changes in the levels of mRNAs encoding c-MET, PI3KC2β, PLCγ1, Cdc42, and ROCK1 in *H. pylori*-infected AGS cells, with and without ASTX pretreatment, determined by real-time PCR. AGS cells were pretreated with vehicle (DMSO) or 5 μM ASTX for 3 h and then incubated with *H. pylori* at a 1:50 cell ratio for 1 h. The relative amounts of c-MET, PI3KC2β, PLCγ1, Cdc42, and ROCK1 mRNA in AGS cells were measured by real-time PCR. The mRNA levels were normalized to that of β-actin (**A**) or 18S rRNA (**B**). All values are expressed as the mean ± S.E. For each experiment, the number of each group was three (*n* = 3 per each group). A Student’s *t*-test was used for statistical analysis. * *p* < 0.05 vs. uninfected cells; + *p* < 0.05 vs. *H. pylori*-infected cells without ASTX treatment.

To compare the transcriptional level of the DEGs encoding c-MET, PI3KC2β, PLCγ1, Cdc42, and ROCK1 in *H. pylori*-infected AGS cells with and without ASTX pretreatment, real-time PCR was carried out. The results reported in Figure 5 show that *H. pylori* infection increases the respective mRNA levels in the absence of ASTX pretreatment, whereas *H. pylori* infection of ASTX-pretreated cells had little or no effect on the mRNA levels. 

## 3. Discussion

Cell migration, which is essential to cancer metastasis, is triggered by coordinated modulation of the cytoskeleton and cell–cell and cell–substratum adhesions. A moving cell extends itself at the front by creating protrusions in the intended direction and by making new focal adhesions with the substratum. On the rear ends of the cell, the cell contracts itself and releases its adhesions from the substratum. *H. pylori* infection triggers the inflammatory response which directly correlates to higher rate of gastric cancer progression by stimulating cell proliferation, migration, angiogenesis, and invasion [44,45,46]. Al-Ghoul et al. investigated the association of cagPAI type IV secretion system in cell motility and IL-8 release, and found out that *H. pylori* that induced high secretion of IL-8 in AGS cells were the ones that are capable of triggering cell motility response in the cells [44]. mRNA expression of IL-8 was notably increased starting from 1 h incubation with *H. pylori*, and IL-8 was affecting most of the early phase signal pathways in AGS cells infected with *H. pylori*, especially around 1 h of infection [47,48]. Major signaling pathways leading to migration and invasion, such as PI3K/Akt, PLCγ, and MAPKs such as p38 and ERK, were activated at early time points of *H. pylori* infection around 30–60 min [47,49,50]. The downstream effectors such as c-Met, Rac1, and Cdc42 were detected in AGS cells at 1 h after *H. pylori* infection [22,51,52]. In this study, preliminary data showed marked increase of IL-8 mRNA expression level from 1 h incubation with *H. pylori*, and thus we aimed to find out which signaling networks are turned on during the first phase response of short-term *H. pylori* infection.

GO analysis of the DEGs identified by RNA-Seq analysis of ASTX-pretreated, *H. pylori*-infected AGS cells, identified 23 DEGs that are associated with cytoskeleton-remodeling, cell-motility, and cell-migration signaling pathways (Figure 3, Table 1). We focused our attention on five of these, namely the c-MET, PI3KC2β, PLCγ1, Cdc42, and ROCK1 encoding genes, and demonstrated that their transcription is upregulated by *H. pylori*, but significantly less so in ASTX-pretreated AGS cells (Figure 5).

The HGF receptor c-Met is known to be an upstream regulator of cell migration [53]. It is highly expressed in cancer cells, where it promotes actin cytoskeleton rearrangement and cell motility [54]. It has also been demonstrated that micro-RNA-499a-mediated suppression of non-small cell lung cancer cell migration and invasion occurs via downregulation of c-MET gene expression [55]. In addition, Churin et al. reported that expression of the c-MET gene is activated within 30 min following *H. pylori* infection of AGS cells, and that this activation is required for *H. pylori*-induced cell motility and scattering [49]. We found that ASTX treatment significantly reduces upregulated c-MET gene expression in *H. pylori*-infected AGS cells (Table 1, Figure 3). Thus, ASTX might target c-Met to suppress AGS cell migration.

The downstream regulators PI3K and PLCγ facilitate the cell motility cascade by modulating the level of phosphatidylinositol 4,5-bisphosphate (PIP2) [56,57,58]. PI3K generates phosphatidylinositol 3,4,5-trisphosphate (PIP3) from PIP2, or generates PIP2 from phosphatidylinositol 4-phosphate (PIP), thereby facilitating Rho GEF activation and membrane targeting. PI3K is an essential mediator of *H. pylori*-induced AGS cell migration [59]. In previous studies, ASTX treatment reduced invasive behavior of breast cancer cells and oral carcinoma cells by inhibiting PI3K signaling [42,43]. In a recent study carried out with oral squamous cell carcinoma models, Kowshik et al. reported that ASTX inhibits hallmarks of cancer by targeting the PI3K/NF-κΒ/STAT3 signaling axis [43]. PLCγ is also an important mediator of cell migration in response to growth factor receptor activation [60,61]. PLCγ modulates the level of PIP2, which in turn governs the activation of Rho family proteins as well as actin-modifying proteins such as the actin-severing cofilin [62]. In the present study, we showed that ASTX suppresses upregulation of the transcription of PI3KC2 and PLCγ1, and their downstream targets ROCK1 and Cdc42 resulting from *H. pylori* infection of AGS cells. 

## 4. Materials and Methods

### 4.1. Cell Line and Culture Conditions

The human gastric adenocarcinoma cell line AGS (ATCC CRL 1739) was purchased from the American Type Culture Collection (ATCC; Rockville, MD, USA). AGS cells were grown in RPMI 1640 medium (Gibco, Grand Island, NY, USA) supplemented with 10% fetal bovine serum (FBS) (Gibco, Grand Island, NY, USA), 100 U/mL penicillin, and 100 μg/mL streptomycin. The cells were cultured at 37 °C under a humidified atmosphere consisting of 95% air and 5% CO_2_.

### 4.2. Treatment of AGS Cells with ASTX

ASTX (Sigma-Aldrich, St. Louis, MO, USA) was dissolved in dimethyl sulfoxide (DMSO) (Sigma-Aldrich, St. Louis, MO, USA) and stored under nitrogen at −80 °C. Before use, the ASTX stock solution was thawed at 70 °C and added to FBS to achieve the desired concentration. Prior to their infection with *H. pylori*, AGS cells were preincubated with pure DMSO diluted by FBS at 0.05% final concentration (v/v) (vehicle control) or with 5 μM ASTX for 3 h. The indicated dose and incubation time of ASTX were chosen based on our previous study that demonstrated the inhibitory effect of ASTX on mitochondrial dysfunction and inflammation in *H. pylori*-infected AGS cells [63]. In particular, 5 μM ASTX exhibited antioxidative effects in AGS cells when preincubated for 3 h.

### 4.3. Bacterial Strain and H. pylori Infection

The *H. pylori* strain NCTC 11637 was obtained from ATCC. The bacterial cells were grown on chocolate agar plates (Becton Dickinson Microbiology Systems, Cockeysvile, MD, USA) at 37 °C, under microaerophilic conditions, using an anaerobic chamber (BBL Campy Pouch^®^ System, Becton Dickinson Microbiology Systems, Franklin Lakes, NJ, USA). AGS cells were seeded and cultured overnight to reach 80% confluency. The *H. pylori* was harvested from the plates, suspended in antibiotic-free RPMI 1640 medium supplemented with 10% fetal bovine serum, and then added to the AGS cell culture at a cellular ratio of 50:1. AGS cells (1.5 × 10^5^/mL) were pretreated with 5 μM ASTX or the control vehicle DMSO for 3 h prior to *H. pylori* stimulation. The cells were then incubated with *H. pylori* for 1 h (for preparation of RNA extracts and for determination of mRNA gene expressions).

### 4.4. Preparation of Total RNA Extracts and Library Construction

Total RNA extract was isolated using TRI reagent (Molecular Research Center, Cincinnati, OH, USA) and then cleaned and concentrated using the RNeasy MinElute Cleanup Kit (Quiagen, Valencia, CA, USA) according to the manufacturer’s protocol. Following determination of the total RNA concentration in each extract, the extracts from three replicate experiments were pooled for RNA-Seq library construction.

AmpliSeq libraries were constructed and sequenced using the Ion Torrent S5^TM^XL next-generation sequencing system (ThermoFisher Scientific, Waltham, MA, USA) in conjunction with the Ion AmpliSeq Transcriptome Human Gene Expression Kit, according to the manufacturer’s instructions. For each sample, 30 ng of total RNA was used for cDNA library preparation. Multiple libraries were multiplexed and clonally amplified using the Ion Chef System (Thermo Fisher Scientific, Waltham, MA, USA), and then sequenced. The ampliSeq RNA Plug in (ver 5.6.0.3) by Torrent Suite Software (Thermo Fisher Scientific, Waltham, MA, USA) was used for data analysis. 

### 4.5. RNA-Sequencing and Bioinformatics Analysis

The total RNA library was subjected to transcriptome sequencing carried out by e-Biogen (www.e-biogen.com, Seoul, Korea). ExDEGA (Excel-based Differentially Expressed Gene Analysis) software (e-Biogen, Seoul, Korea) was used for initial data processing and analysis for differentially expressed genes (DEGs). 

Differential gene expression analysis was performed with ExDEGA v1.6.8 software and using a cutoff at the normalized gene expression (log2) of 2 and *p*-value of <0.05. DEGs were identified based on >1.5-fold change observed in transcript levels. After filtering the DEGs, Gene Ontology (GO) analysis was performed using the DAVID bioinformatics program (https://david.ncifcrf.gov), for gene identification and annotation. To identify the functional groups and molecular pathways associated with the observed DEGs, RNA-Seq data were further analyzed using the Kyoto Encyclopedia of Genes and Genomes (KEGG) database (www.genome.jp).

### 4.6. Validation of DEGs by Real-Time Polymerase Chain Reaction (PCR)

Real-time PCR was performed to validate the expression profiles of DEGs identified from RNA-Seq analysis. Candidate genes were selected in relation to the category of function. Total RNA was isolated using TRI reagent (Molecular Research Center, Cincinnati, OH, USA). Conversion of the RNA to cDNA was carried out by incubating the RNA sample with a random nucleotide hexamer and MuLV reverse transcriptase (Promega, Madison, WI, USA) at 23 °C for 10 min, 37 °C for 60 min, and 95 °C for 5 min. The cDNA was used for real-time PCR with primers specific for human. The sequences of the primers used are 5′-TGCACAGTTGGTCCTGCCATGA-3′ (forward) and 5′-CAGCCATAGGACCGTATTTCGG-3′ (reverse) for c-MET, 5′-TTGTCTGTCACACTTCTGTAGTT-3′ (forward) and 5′-AACAGTTCCCATTGGATTCAACA-3′ (reverse) for PI3KC2β, 5′-TCGTATATCAGCCAAGGACC-3′ (forward) and 5′-AGTACTGGCTTCCAAGAAGG -3′ (reverse) for PLCγ1, 5′-GATGGTGCTGTTGGTAAA-3′ (forward) and 5′-TAACTCAGCGGTCGTAAT-3′ (reverse) for Cdc42, 5′-ATGAGTTTATTCCTACACTCTACCACTTTC-3′ (forward) and 5′-TAACATGGCATCTTCGACGACACTCTAG-3′ (reverse) for ROCK1. For PCR amplification, the cDNA was amplified by 45 repeat denaturation cycles at 95 °C for 30 s, annealing at 55 °C for 30 s, and extension at 72 °C for 30 s. During the first cycle, the 95 °C step was extended to 3 min. The β-actin and 18S rRNA gene was amplified in the same reaction to serve as the reference gene. For β-actin and 18S rRNA, the desired PCR product was obtained using the primer 5′-ACCAACTGGGACGACATGGAG-3′ (forward) and 5′-GTGAGGATCTTCATGAGGTAGTC-3′ (reverse), and 5′-GTAACCCGTTGAACCCCATT-3′ (forward) and 5′-CCATCCAATCGGTAGTAGCG-3′ (reverse), respectively. The relative gene expression of c-MET, PI3KC2β, PLCγ1, Cdc42, and ROCK1 mRNA were normalized to that of β-actin or 18S rRNA. 

### 4.7. Statistical Analysis

For the changes in the levels of mRNAs encoding c-MET, PI3KC2β, PLCγ1, Cdc42, and ROCK1 in *H. pylori*-infected AGS cells, all values are expressed as the mean ± S.E. For each experiment, the number of each group was three (*n* = 3 per each group). A Student’s *t*-test was used for statistical analysis. A *p*-value of 0.05 or less was considered statistically significant. 

## 5. Conclusions

Transcriptional array profiling by RNA-Seq analysis of *H. pylori*-infected AGS cells, and *H. pylori*-infected AGS cells pretreated with ASTX was carried out to identify potential mediators in the inhibitory mechanism of ASTX on *H. pylori*-induced inflammatory and carcinogenic responses. Our findings indicate that ASTX can suppress *H. pylori*-induced gastric cancer progression by inhibiting cytoskeleton reorganization and reducing cell motility through downregulation of c-MET, EGFR, PI3KC2, PLCγ1, Cdc42, and ROCK1. 

## Figures and Tables

**Table 1:** Expression levels of DEGs associated with cell migration/motility, which are upregulated in AGS cells in response to *H. pylori* infection but experience less, or no upregulation, in infected cells pretreated with ASTX. The expression level of each gene is reported as the read count normalized to the log2 value.

Gene ID	Normalized (log2) Read Count				Encoded Protein
	None	HP	ASTX	ASTX + HP	
ARF4	7.424	8.152	8.192	6.187	ADP-ribosylation factor 4
CCL20	0.116	2.928	0.000	2.179	Chemokine C-C motif ligand 20
CDC42	9.142	9.921	9.389	6.982	Cell division control protein 42 homolog
CXCL1	1.005	5.432	1.348	4.080	Chemokine C-X-C motif ligand 1
CXCL2	2.008	5.274	2.124	4.697	Chemokine C-X-C motif ligand 2
CXCL3	2.023	5.557	2.193	4.950	Chemokine C-X-C motif ligand 3
FAT1	7.348	8.019	8.158	5.615	FAT atypical cadherin 1
FUT8	6.201	7.140	7.199	6.646	Fucosyltransferase 8 alpha 1,6 fucosyltransferase
GSK3A	7.385	8.294	8.267	8.362	Glycogen synthase kinase 3 alpha
ITGA1	1.946	2.266	1.831	1.728	Integrin alpha 1
IL8	0.704	7.118	0.341	6.954	Interleukin 8
MET	5.712	6.961	6.059	6.022	Met proto-oncogene
NDE1	5.108	5.734	5.819	5.165	Nude neurodevelopment protein 1
PIK3CB	5.937	7.222	6.233	4.757	Phosphatidylinositol-4,5-bisphosphate 3-kinase catalytic subunit beta
PKN2	4.999	5.727	5.828	4.947	Protein kinase N2
PLCG1	6.315	6.806	6.962	6.451	Phospholipase C gamma-1
RND1	3.343	4.082	3.228	3.647	Rho family GTPase 1
ROCK1	4.122	4.820	4.957	4.123	Rho-associated coiled-coil containing protein kinase 1
SEMA7A	2.916	3.803	3.406	2.948	Semaphoring 7A GPI membrane anchor
SLC9B2	2.137	2.614	2.601	2.130	Solute carrier family 9 subfamily B2
TNFRSF10B	6.533	7.260	6.763	3.900	Tumor necrosis factor receptor superfamily member 10b
